# Renal Function Improvement Following ANG-3777 Treatment in Patients at High Risk for Delayed Graft Function After Kidney Transplantation

**DOI:** 10.1097/TP.0000000000003255

**Published:** 2021-01-26

**Authors:** Jonathan S. Bromberg, Matthew R. Weir, A. Osama Gaber, Michael A. Yamin, Itzhak D. Goldberg, Tracy J. Mayne, Weizhong Cal, Matthew Cooper

**Affiliations:** 1 Division of Transplantation, Department of Surgery, Western Maryland Regional Medical Center, University of Maryland, Baltimore, MD.; 2 Department of Surgery, Houston Methodist Hospital, J.C. Walter Jr. Transplant Center, Houston, TX.; 3 Angion Biomedica, New York, NY.; 4 Medstar Georgetown Transplant Institute, Washington, DC.

## Abstract

**Background.:**

Patients (20%–50%) undergoing renal transplantation experience acute kidney injury resulting in delayed graft function. ANG-3777 is an hepatocyte growth factor mimetic that binds to the c-MET receptor. In animal models, ANG-3777 decreases apoptosis, increases proliferation, and promotes organ repair and function.

**Methods.:**

This was a randomized, double-blind, placebo-controlled, phase 2 trial of patients undergoing renal transplantation with <50 cc/h urine output for 8 consecutive hours over the first 24 hours posttransplantation, or creatinine reduction ratio <30% from pretransplantation to 24 hours posttransplantation. Subjects were randomized as 2:1 to 3, once-daily IV infusions of ANG-3777, 2 mg/kg (n = 19), or placebo (n = 9). Primary endpoint: time in days to achieve ≥1200 cc urine for 24 hours.

**Results.:**

Patients treated with ANG-3777 were more likely to achieve the primary endpoint of 1200 cc urine for 24 hours by 28 days posttransplantation (83.3% versus 50% placebo; log-rank test: χ^2^ = 2.799, *P* = 0.09). Compared with placebo, patients in the ANG-3777 arm had larger increases in urine output; lower serum creatinine; greater reduction in C-reactive protein and neutrophil gelatinase-associated lipocalin; fewer dialysis sessions and shorter duration of dialysis; fewer hospital days; significantly less graft failure; and higher estimated glomerular filtration rate. Adverse events occurred in a similar percentage of subjects in both arms. Events per subject were twice as high in the placebo arm.

**Conclusions.:**

There was an efficacy signal for improved renal function in subjects treated with ANG-3777 relative to placebo, with a good safety profile.

## Abstract 2

Supplemental Digital Content is available in the text.

## INTRODUCTION

Kidney transplantation improves health outcomes and quality of life at a reduced cost relative to dialysis.^1,2^ With approximately 100 000 patients on the kidney transplant waitlist in the United States, but only approximately 20 000 kidney transplants performed each year, it is critical that renal transplantation and graft survival be maximized.^3^

The 2 leading contributors to early graft failure are acute rejection and acute kidney injury (AKI) occurring during the transplantation process.^4,5^ While rejection can be managed utilizing immunosuppressant therapy, no therapies currently exist for delayed graft function (DGF)–associated AKI. Leading factors that contribute to DGF-associated AKI include donor injury, length of cold ischemia time, and ischemia-reperfusion injury, which are significantly higher in organs procured from deceased than living donors.^4^ The histologic and morphologic features of AKI include effacement and loss of proximal tubule brush border, patchy loss of tubule cells, focal areas of proximal tubular dilation and distal tubular casts, and apoptotic cell death in proximal and distal tubules.^6-8^ Transplantation-associated AKI can result in primary graft nonfunction or DGF.^9-11^ While there are several definitions of DGF, one of the most commonly accepted is the need for renal replacement therapy in the first week after transplantation.^12^ Most centers report a DGF rate of 20%–50%.^13^ Kidneys that manifest DGF have a higher frequency of adverse patient outcomes, including decreased graft function (eg, increased SCr/decreased eGFR), decreased graft survival, and increased mortality.^14-17^ In a recent meta-analysis, the DGF hazard ratio for 1-year graft loss was 1.89 (95% CI = 1.46-2.47).^18^ Increased risk of DGF also leads to rejection of organs deemed less viable, suppressing maximization of transplanting procured organs.^19^

When DGF is diagnosed, the main management strategies are supportive care, including dialysis and monitoring for rejection with serial biopsies. DGF is associated with increased medical costs, including longer hospital stays, more frequent outpatient visits, increased imaging, increased invasive procedures including dialysis, and pharmacologic therapies.^20-22^

ANG-3777 is a small molecule hepatocyte growth factor (HGF) mimetic currently under clinical development for the treatment of AKI in renal transplant patients with signs of DGF. The biologic effects of HGF are mediated by a signal cascade initiated by binding of HGF to its transmembrane tyrosine kinase receptor, c-MET.^23-25^ c-MET is expressed on epithelial cells of most tissues as well as on endothelial cells, vascular smooth muscle cells, microglial cells, neurons, and cardiac myocytes. c-MET is upregulated in the setting of acute tissue injury, with peak receptor expression occurring approximately 24 hours after injury.^26,27^ HGF is endogenously released within minutes after injury, with peak serum concentration achieved at approximately 2 hours.^28^ Thus, there is a mismatch between peak serum HGF concentration and peak c-Met receptor availability, representing a potential window around 24 hours for optimizing c-Met activation through the administration of an HGF mimetic. Interaction of HGF and c-MET activates cellular pathways leading to a reduction in apoptosis and an increase in proliferation and regeneration. In *in vivo* models of renal injury secondary to ischemia or toxin administration, HGF treatment reduced tubular necrosis, decreased renal epithelial apoptosis, and augmented renal regeneration.^29,30^ In vitro studies of ANG-3777 in multiple cell lines have shown that it induces c-MET dimerization and phosphorylation, reducing apoptosis and increasing cellular proliferation.^31,32^ In animal models of renal injury (including nephrotoxic, ischemia–reperfusion, and transplantation), exposure to ANG-3777 at 24 hours after acute renal injury reduced both renal dysfunction and mortality.^33-35^

The objective of this study was to evaluate the safety and efficacy of ANG-3777 in subjects who have undergone kidney transplantation and had signs of AKI, placing them at high risk for dialysis.

## MATERIALS AND METHODS

This method was a multicenter, randomized, double-blind, placebo-controlled, phase 2 trial designed as a signal detection study for ANG-3777 for the treatment of DGF. The population of patients undergoing kidney transplantation was enriched for high risk of DGF as having either oliguria or low creatinine reduction ratio (CRR) in the first 24 hours posttransplantation. In previous research, patients producing <50 cc/h urine output for 6 consecutive hours postrenal transplantation were 13 times more likely to require dialysis than those without oliguria.^36^ A CRR <30% within 48 hours after transplant has been shown to have equivalent negative clinical outcomes to patients who receive dialysis in the first week.^37^ To enrich the sample, patients were enrolled who produced <50 cc urine for 8 consecutive hours in the first 24 hours posttransplantation, and CRR <30% from pretransplantation to 24 hours posttransplantation. In addition, the kidney donor had to have a terminal creatinine ≤2.2 mg/dL.

Eligible subjects were randomized 2:1 to receive 3 administrations of ANG-3777, 2 mg/kg via 30 minutes intravenous infusion, or placebo. The initial infusion occurred 24–36 hours after the transplantation, with 2 subsequent infusions at 24-hour (±2 h) intervals. Infusion volume was controlled via an infusion pump based upon the subject’s baseline weight (0.33 mL/kg), with a maximum infusion of 40 mL. Placebo was normal saline administered at the same intervals and volumes.

Subjects were monitored during initial hospitalization, with subsequent clinical visits on days 7, 14, and 28, and phone contact on days 5, 6, 8, and 13. Twenty-four-hour urine was collected at home after initial hospitalization. Long-term data on graft survival and renal function were collected 6 and 12 months after transplantation.

During hospitalization, blood samples were collected to assess immunosuppressant trough levels. Daily 24-hour urine output was assessed for 14 days after the first infusion and again on day 28. Other assessments included standard safety measures, blood and urine biomarkers of renal function, and 24-hour urine creatinine clearance.

Patients recruited were males and females aged ≥18 years receiving a renal transplantation because of end-stage renal disease requiring long-term dialysis, whose urine output was <50 cc/h for >8 consecutive hours, or with a CRR <30% from pretransplantation to 24 hours posttransplantation. Patients with preemptive renal transplantation, multiple organ transplantation, and cold ischemia time >40 hours were excluded. A complete list of inclusion and exclusion criteria can be found in the supplemental digital content (SDC1, http://links.lww.com/TP/B923).

The study was conducted in accordance with the Guideline for Good Clinical Practice (CPMP/ICH/135/95—July 17, 1996) and in full conformity with the regulations and guidelines of the US FDA and under approval from the institutional review board of each participating institution.

### Efficacy Measures and Analysis

The primary efficacy measure was time (in d) from transplantation to production of ≥1200 cc of urine for a 24-hour period, based upon the research demonstrating that this degree of oliguria is highly correlated with subsequent graft function.^36^ The primary efficacy analysis was a log-rank test comparing the Kaplan–Meier time to event curves between ANG-3777 and placebo. The median number of days to achieve ≥1200 cc urine output for 24 hours and 95% CI were computed for each group. Power was not prespecified.

Secondary efficacy measures were analyzed descriptively. Mixed models repeated measures (MMRM) were used to calculate the least square (LS) means and standard errors (SEs) by timepoint. Secondary endpoints included:

- total daily urine output: days 1–14
- change from baseline urine production: days 2–14
- SCr: days 4, 7, 10, 14, 28
- measured 24-hour creatinine clearance: days 3, 7, 14, and 28
- serum C-reactive protein (CRP) (marker of general inflammation) and neutrophil gelatinase-associated lipocalin (NGAL, marker of tubular damage): days 1 and 3
- incidence of DGF (ie, dialysis within the first 7 d posttransplantation)
- number of dialysis sessions: days 1–28
- length of transplantation hospitalization
- acute rejection through 12 months

In response to the March 2017 Food and Drug Administration (FDA) Draft Guidance for Industry on Delayed Graft Function in Kidney Transplantation, 3 post hoc analyses were conducted.^38^ The draft guidance specified that duration of dialysis, 12-month eGFR, and graft failure are acceptable efficacy endpoints for DGF trials. The sponsor and FDA are in discussion regarding the inclusion of these endpoints in the phase 3 study. The post hoc analyses included:

- log-rank test of time to graft failure during the first 12 months after transplantation by study arm
- MMRM of mean eGFR at screening, day 3, day 7, day 14, day 28, month 6, and month 12 by study arm. eGFR was derived from SCr using the modification of diet in renal disease 4 variable equation.^39^
- descriptive analysis of duration of dialysis during the first 28 days posttransplantation by study arm

For the 2 placebo patients with graft failure, eGFR was prespecified to be set to zero after failure. For both SCr and eGFR, sensitivity analyses were run setting eGFR to 10 mL/min/1.73 m^2^ and SCr to 7 mg/dL, as utilized by the National Institutes of Health Clinical Trials on Transplant group.^40^ Additional sensitivity analysis excluded SCr and eGFR values for graft failure patients after failure.

### Safety Analysis

Safety analyses included incidence of adverse events (AEs), treatment-emergent AEs (TEAEs), treatment-related TEAEs, TEAEs rated by severity (mild, moderate, and severe), serious AEs (SAEs), treatment-emergent SAEs (TESAEs), and treatment-related SAEs. AE summary tables include the number and percentage of subjects experiencing an AE and number of events of an AE. Descriptive statistics for hematology and chemistry results were calculated for each time point.

## RESULTS

### Patient Characteristics

Twenty-nine subjects were screened and consented to participate at 5 US sites. One subject was judged by their physician as too fragile to participate and was withdrawn before randomization. The remaining 28 subjects were randomized: 19 to ANG-3777 and 9 to placebo. One subject in the placebo arm withdrew consent after the second infusion. The withdrawal was unrelated to study product. Based on a review of an unplanned interim analysis of the first 20 patients, the FDA agreed that a sufficient efficacy signal had been demonstrated to allow for the initiation of a phase 3 trial. At the time of FDA review, 28 subjects had been enrolled and further recruitment was halted.

Subject characteristics are shown in Table 1. Subjects in the ANG-3777 arm were younger (54.7 ± 13.7 versus 65.7 ± 12.8 y), more likely to be female (21.1% versus 11.1%), more likely to be black/African American (42.1% versus 22.2%), and had higher body weight, dry weight, and BMI.

**TABLE 1.** Subject characteristics by study arm

	ANG-3777 (N = 19)	Placebo (N = 9)
Age (y), mean (SD)	54.7 (13.7)	65.7 (12.87)
Gender, n (%)		
Male	15 (78.9)	8 (88.9)
Female	4 (21.1)	1 (11.1)
Race, n (%)		
White	9 (47.4)	5 (55.6)
Black or African American	8 (42.1)	2 (22.2)
Asian	1 (5.3)	1 (11.1)
American Indian or Alaska Native	1 (5.3)	0 (0.0)
Other	0 (0.0)	1 (11.1)
Body weight (kg), mean (SD)	90.9 (17.1)	79.8 (16.2)
Dry weight (kg), mean (SD)	86.9 (16.6)	80.9 (18.0)^*a*^
BMI (kg/m^2^), mean (SD)^*b*^	30.1 (4.67)	28.0 (4.0)
Medical history, n (%)		
Hypertension	18 (94.7)	9 (100.0)
Diabetes and hyperglycemia	13 (68.4)	6 (66.7)
Cardiovascular	15 (78.9)	9 (100.0)
Gastrointestinal	10 (52.6)	5 (55.6)
Musculoskeletal	7 (36.8)	4 (44.4)
Other surgeries	7 (36.8)	4 (44.4)
Neuropsychiatric	5 (26.3)	5 (55.6)
Urogenital	6 (31.6)	3 (33.3)
Liver	5 (26.3)	3 (33.3)
Respiratory	4 (21.1)	3 (33.3)
Eye	5 (26.3)	2 (22.2)

^*a*^In the placebo arm, mean dry weight exceeded mean body weight because of the incomplete dry weight data (n = 9 for wet body weight, n = 6 for dry weight).

^*b*^Body weight was used for calculation of body mass index.

BMI, body mass index.

Hypertension was nearly universal (ANG-3777 = 94.7%; placebo = 100.0%). Two-thirds of subjects in both arms had a history of diabetes/hyperglycemia. Cardiovascular disease was higher in placebo versus ANG-3777 (100.0% versus 78.9%) because of a higher prevalence of atherosclerotic disease/procedures, arrhythmia, and valve disease/disorder. The incidence of neuropsychiatric and respiratory disorders was higher in the placebo arm (55.6% versus 26.3% and 33.3% versus 21.1%, respectively) but did not cluster by a specific diagnosis. Groups were otherwise similar in disease history.

Table 2 shows donor and transplantation characteristics. Most kidneys were from donors after brain death (ANG-3777 = 68.4%; placebo = 77.8%). However, there were more donors after circulatory death in the ANG-3777 arm (21.1%; versus 11.1%). Donors in the ANG-3777 arm were younger than placebo (43.0 ± 21.2 versus 56.3 ± 10.8 y) and had lower prevalence of diabetes/hypertension (ANG-3777 = 63.2%; placebo = 77.8%). Mean time from organ procurement to transplantation was similar (ANG-3777 = 23.3 ± 9.2; placebo = 23.7 ± 10.3 h) as was mean transplantation time (ANG-3777 = 3.2 ± 1.6; placebo = 3.0 ± 0.9 h).

**TABLE 2.** Donor and transplantation characteristics

	ANG-3777 (N = 19)	Placebo (N = 9)
Donation type, n (%)		
Donation after brain death	13 (68.4)	7 (77.8)
Donation after circulatory death	4 (21.1)	1 (11.1)
Live	2 (10.5)	0
Unknown	0	1 (11.1)
History of diabetes mellitus or hypertension		
Yes	12 (63.2)	7 (77.8)
No	7 (36.8)	2 (22.2)
Donor age (y), mean (SD)	43.0 (21.2)	56.3 (10.8)
H from organ procurement to transplantation, mean (SD)	23.3 (9.2)	23.7 (10.3)
Total transplantation time (h), mean (SD)	3.2 (1.6)	3.0 (0.9)
Irish nomogram risk score, mean (SD)	49.6% (3.2)	51.0% (2.5)

To assess potential imbalances in baseline risk, we compared study arms on factors shown by Irish et al to produce a comprehensive risk score highly predictive of DGF, known as the Irish nomogram.^41^ The arms were equivalent in risk of DGF (ANG-3777 mean = 49.6% ± 3.2; placebo mean = 51.0% ± 2.5). Thus, the percentage of subjects receiving any dialysis in the first 7 days was very similar between groups (ANG-3777 = 73.6%; placebo = 66.6%). Note, 10 of 14 (71%) subjects with DGF in the ANG-3777 arm had their first dialysis session on day 1, before study drug was administered, versus 2 of 6 (33%) in the placebo arm.

### Efficacy Results

#### Primary Endpoint

**FIGURE 1.**
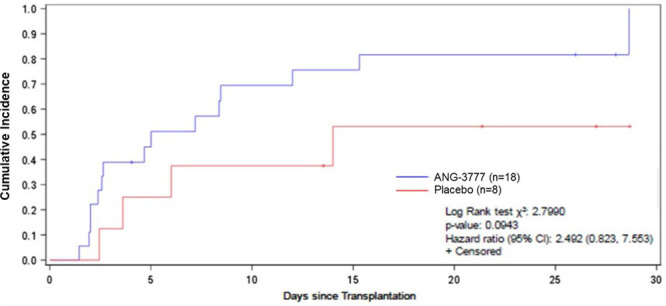
Time to production of ≥1200 cc urine over 24 h by study arm.

Figure 1 shows Kaplan–Meier curves for time to production of ≥1200 cc urine for 24 hours by study arm. One subject in each study arm reached this endpoint before the start of the first infusion and was excluded from this analysis but was included in all other analyses. At day 28, 83.3% of subjects in the ANG-3777 arm achieved >1200 cc urine output for 24 hours versus 50.0% in placebo; time to event was shorter in the ANG-3777 arm (log-rank test: χ^2^ = 2.799, *P* = 0.094; hazard ratio [HR] = 2.49, 95% CI = 0.82-7.55). The median number of days from transplantation to the production of ≥1200 cc of urine for 24 hours was 5 for ANG-3777 (95% CI = 2.4-12.0) and 14 for placebo (95% CI = 2.44-). The placebo upper limit could not be calculated as only 50% of subjects achieved the outcome.

#### Secondary Endpoints

**FIGURE 2.**
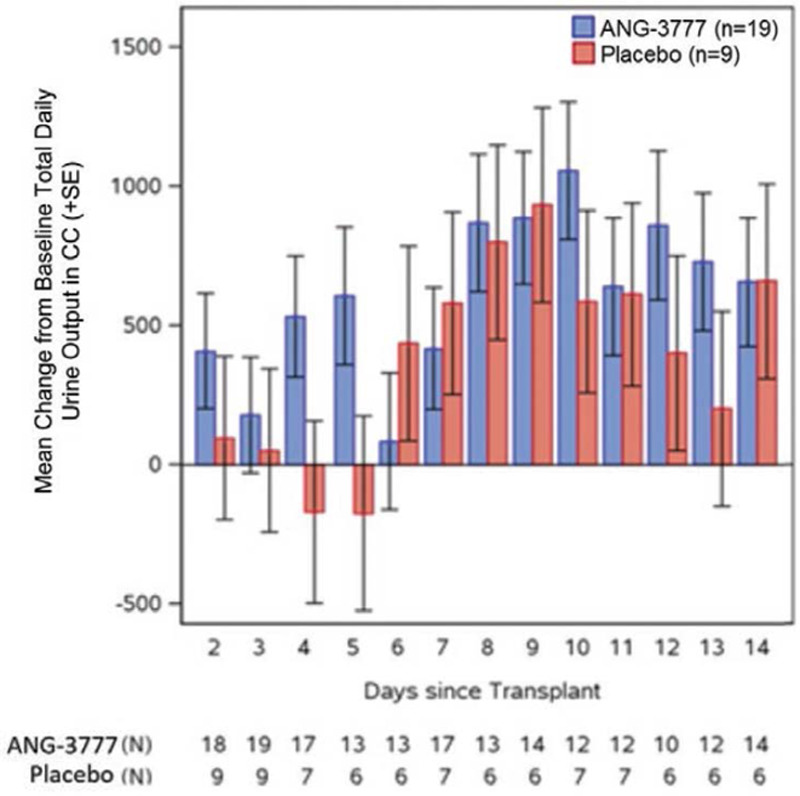
Change from baseline urine production d 2–14 by study arm.

Day 1 posttransplantation urine production was higher in the ANG-3777 arm (690 ± 727 cc) versus placebo (600 ± 897 cc). Figure 2 shows MMRM LS means and SEs for the change from day 1 total daily urine output to days 2–14 by study arm. On days 2–5, the ANG-3777 arm showed increases from baseline urine production (d 2 = +408, d 3 = +178, d 4 = +531, and d 5 = +606); the placebo arm showed small increases or decreases (d 2 = +95, d 3 = +51, d 4 = –171, and d 5 = –176). On days 6 and 7, the ANG-3777 arm showed a smaller increase from baseline than placebo (d 6: +83 versus +436; d 7: +417 versus +579). On days 8, 10, 12, and 13, the change from baseline total daily urine production was greater in the ANG-3777 arm versus placebo (d 8: +869 versus +799; d 10: +1056 versus +585; d 12: +860 versus +400; d 13: +729 versus +200). The differences between study arms were <50 cc on days 9, 11, and 14.

**FIGURE 3.**
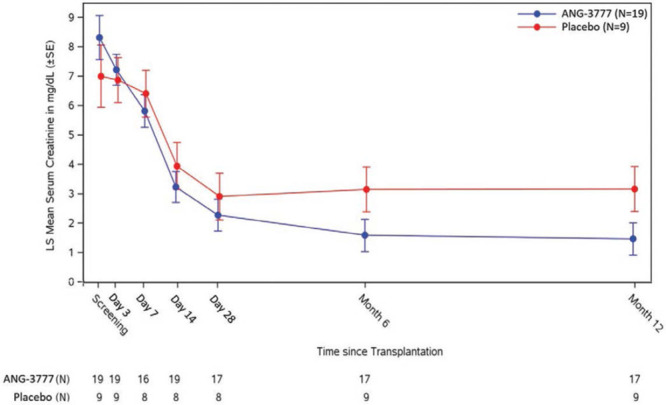
Serum creatinine over time by study arm.

Figure 3 shows MMRM LS means and SEs for SCr (mg/dL) during time by study arm. Subjects in the ANG-3777 arm had higher SCr versus placebo at screening (difference = +1.31 mg/dL) and day 3 (difference = +0.34 mg/dL), and lower SCr on days 7, 14, 28, month 6, and month 12 (difference = –0.59, –0.71, –0.63, –1.56, and –1.70 mg/dL, respectively). If values for SCr are removed for patients with graft failure, the differences between arms at 6 and 12 months are attenuated but continue to favor ANG-3777 (difference: d 7 = –0.67, d 14 = –0.79, d 28 = –0.70, mo 6 = –0.29, mo 12 = –0.43 mg/dL). Creatinine clearance could not be analyzed because of excessive missing data.

Day 1 CRP was higher in the ANG-3777 arm (LS mean = 7.5, SE = 0.9 mg/dL) versus placebo (LS mean = 5.0, SE = 1.8 mg/dL). On day 3, CRP was lower in the ANG-3777 arm (LS mean = 2.2, SE = 0.4) versus placebo (LS mean = 2.9, SE = 0.7). The mean within-subject decrease in CRP was greater in the ANG-3777 arm (–68.6% from d 1) than in placebo (–17.5%). Day 1 NGAL was higher in the ANG-3777 arm (LS mean = 1152, SE = 121) versus placebo (LS mean = 646, SE = 102). On day 3, NGAL was nearly equivalent between arms (ANG-3777: LS mean = 614, SE = 85; placebo: LS mean = 571; SE = 101). There was a greater mean within-subject decrease in NGAL in the ANG-3777 arm (–43.5% from baseline) than in placebo (–12.5%).

The mean number of dialysis sessions through day 28 posttransplantation was higher in the placebo arm (LS mean = 3.8, SE = 1.4) versus ANG-3777 (LS mean = 2.8, SE = 0.6). The LS mean duration of dialysis was 2.4 days shorter for the ANG-3777 arm (LS mean = 7.6, SE = 2.0) versus placebo (LS mean = 10.0, SE = 3.9). The LS mean length of hospitalization was 7.6 days (SE = 0.5) for ANG-3777 and 11.4 days (SE = 3.4) for placebo, a difference of 3.8 days. There were no episodes of acute rejection in either study arm through day 28.

#### Post Hoc Analyses

**FIGURE 4.**
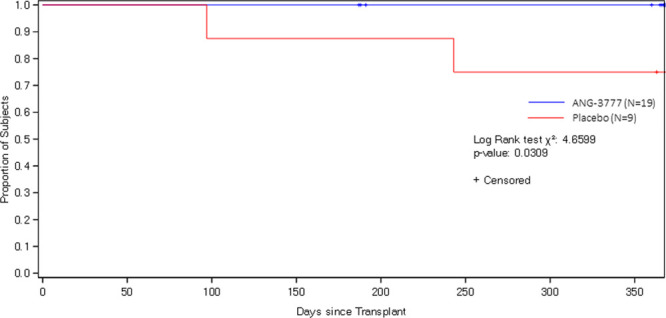
Time to graft failure by study arm.

Figure 4 shows the incidence of graft failure over time by study arm. Two subjects (22.2%) in the placebo arm experienced graft failure within the first 12 months after transplantation, versus no subjects in the ANG-3777 arm (χ^2^ = 4.66, *P* = 0.03).

There was a significant correlation between eGFR measures over time. A Pearson product-moment correlation matrix showed that from day 28 onward, all correlations exceeded r = 0.50, with the highest correlation between 6- and 12-month eGFR (r = 0.84). Therefore, an autoregressive covariance structure was used for the eGFR MMRM.

**FIGURE 5.**
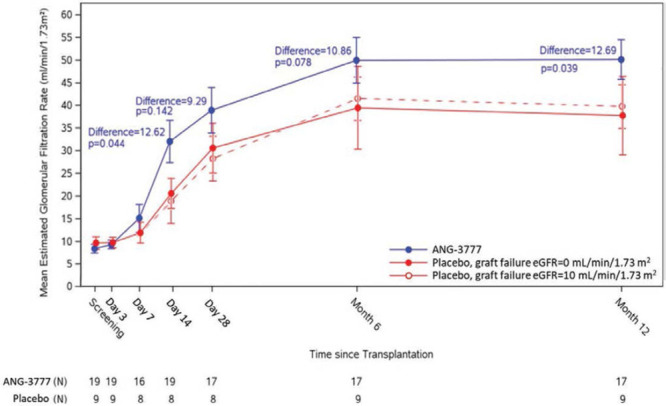
Estimated glomerular filtration rate over time by study arm.

As shown in Figure 5, eGFR (in mL/min/1.73 m^2^) was higher in the ANG-3777 arm versus placebo arm on day 14 (LS mean = 32.3, SE = 3.4 versus LS mean = 19.7, SE = 5.2; *P* = 0.04), day 28 (LS mean = 38.9, SE = 3.6 versus LS mean = 29.6, SE = 5.2; *P* = 0.14), month 6 (LS mean = 49.9, SE = 3.6; versus LS mean = 39.1, SE = 5.0; *P* = 0.08), and month 12 (LS mean = 50.0, SE = 3.6; versus LS mean = 37.3, SE = 5.0; *P* = 0.04). The absolute differences among groups at day 14, day 28, month 6, and month 12 were 12.6, 9.3, 10.9, and 12.7 mL/min/1.73 m^2^, respectively. When eGFR for subjects with graft failure was set to 10 in mL/min/1.73 m^2^ as (dashed line), the effect was slightly attenuated at 6 and 12 months, with differences of 7.9 and 10.3 mL/min/1.73 m^2^. When values for eGFR are removed from the model for patients with graft failure, the differences among arms on day 14, day 28, month 6, and month 12 were attenuated but persisted (differences = 13.3, 10.1, 1.4, and 4.6 mL/min/1.73 m^2^, respectively).

### Safety Results

#### Adverse Events

There were no deaths or discontinuations of study drug because of AEs. Table 3 presents an overview of AEs by study arm. In the ANG-3777 arm, there was a total of 99 AEs reported in 17 subjects (89.5%), an average of 5.8 events per subject. In the placebo arm, there were 89 AEs reported in 8 subjects (88.9%), an average of 11.1 events per subject. TEAEs followed a similar pattern. In the ANG-3777 arm, 83 TEAEs were reported in 15 subjects (78.9%), an average of 5.5 TEAEs per subject. In the placebo arm, 78 TEAEs were reported in 8 subjects (88.9%), an average of 9.8 TEAEs per subject. Six of these TEAEs in the ANG-3777 arm, occurring in 3 subjects, were assessed by the investigator as related to study drug: 2 infusion site reactions in one subject, 2 instances of nausea and vomiting in one subject, and 1 instance of decreased blood phosphorus and potassium in one subject. None of the TEAEs in the placebo arm was assessed by the investigator as related to study drug.

**TABLE 3.** Adverse events overview

Subjects with	ANG-3777 (N = 19)		Placebo (N = 9)	
	n (%)	Events	n (%)	Events
≥1 AE	17 (89.5)	99	8 (88.9)	89
≥1 TEAE^*a*^	15 (78.9)	83	8 (88.9)	78
TEAEs related to study drug^*b*^	3 (15.8)	6	0	0
≥1 severe TEAE	6 (31.6)	11	1 (11.1)	1
≥1 SAE	8 (42.1)	16	4 (44.4)	17
≥1 TESAE	8 (42.1)	16	4 (44.4)	17
With TESAEs related to study drug	0	0	0	0
Deaths – all causes	0	0	0	0

^*a*^An adverse event (AE) was considered treatment emergent if the date of onset was on or after the date of the first dose of study treatment through 30 d after the last dose of study treatment, or, if applicable, those with onset before the first administration of study medication but that worsened during the therapy.

^*b*^Related = possibly related, probably related, or definitely related to study drug.

In 7 of the 12 System Organ Classes (SOCs) with TEAEs in >5% of subjects, the event rate in the placebo arm exceeded that in the ANG-3777 arm by >10% (Table S1, SDC, http://links.lww.com/TP/B923). This was largely driven by higher dyspnea and edema in the placebo arm. In the one System Organ Class in which the event rate in the ANG-3777 arm exceeded the placebo arm (renal and urinary disorders), the difference was due to higher nocturia in the ANG-3777 arm (N = 2).

There were 11 TEAEs in the ANG-3777 arm rated as severe versus 1 in the placebo arm. All severe TEAEs were included as SAEs. All SAEs were treatment emergent. In the ANG-3777 arm, 8 subjects (42.1%) reported a total of 16 TESAEs, an average of 2.0 TESAEs per subject. In the placebo arm, 4 subjects (44.4%) reported 17 TESAEs, an average of 4.3 TESAEs per subject. None of the TESAEs in either study arm was assessed by the investigator as related to study drug. The most common System Organ Class for TESAEs was Renal and Urinary Disorders, which included 2 incidents of acute renal failure in the placebo group (Table S2, SDC, http://links.lww.com/TP/B923). Blood chemistry values were frequently out of range in both groups, consistent with postrenal transplantation, and were generally similar between study arms.

Physical examinations and vital signs were mostly within normal ranges, with no clear differences between groups or trends during time. Few subjects had abnormal or clinically significant ECG diagnosis. Increases to ≥500 ms for QTcB were observed in 5 ANG-3777 subjects (26%) and 3 placebo subjects (33%), and for QTcF in 3 ANG-3777 subjects (16%) and 2 placebo subjects (22%). These increases were transient and not considered clinically significant except in one ANG-3777 subject. That ANG-3777 subject consistently had clinically significant abnormal ECG findings because of a medical history of nonischemic cardiomyopathy.

## DISCUSSION

In their totality, efficacy measures showed a pattern supporting a signal for improved short- and long-term kidney function in subjects who received ANG-3777 after transplantation compared with placebo. Safety was similar between groups.

Prerandomization subjects in both arms had significant oliguria 24 hours after transplantation. Whether measured as total urine output, increase in urine output, or reaching a threshold of ≥1200 cc urine for 24 hours, oliguria was more likely to resolve, and resolve more quickly, in subjects who received ANG-3777. Early measures of SCr and derived eGFR were better in the ANG-3777 arm on days 14 and 28. Measures of inflammation (CRP) and kidney tubular damage (NGAL) were higher in the ANG-3777 arm the day after transplantation, but showed greater improvement relative to placebo on day 3. Safety measures provide data supporting enhanced efficacy. Nocturia occurred as an AE in 2 ANG-3777 subjects versus no placebo subjects, which could be interpreted as an efficacy signal given the initial oliguria. Although not shown here, serial assessments of albumin, calcium, and sodium all showed greater improvement in the ANG-3777 arm versus placebo. Incidence of DGF was slightly higher in the ANG-3777 arm, but most subjects had their first dialysis session before study drug was administered. All treatment measures, including the number of dialysis sessions, duration of dialysis, and length of hospital stay, were lower in the ANG-3777 arm.

Long-term measures showed the improvements observed in the ANG-3777 arm during the first 28 days were sustained. Six- and 12-month SCr were lower in the ANG-3777 arm than in the placebo, which translated into, respectively, 11–13 mL/min/1.73 m^2^ higher eGFR in the ANG-3777 arm versus placebo. There were 2 graft failures out of 9 patients in the placebo arm versus none in the ANG-3777 arm. Taken together, the data suggest an efficacy signal for ANG-3777 in patients with signs of kidney injury after transplantation.

There are some important limitations. This was a small study with 19 subjects receiving active treatment and 9 receiving placebo. As a result, the interpretation of efficacy results should be considered directional and not overreliant on significance values. An unplanned interim analysis was conducted after enrollment of 20 subjects, which compromised the ability to draw conclusions based on statistical testing. While there were imbalances between groups on individual baseline risk factors, the overall risk as defined by the Irish nomogram was nearly equivalent, resulting in a similar incidence of DGF. However, this cannot rule out the possibility that a difference between groups, such as donor age, could have affected the observed outcomes. In addition, the goal of this study was to detect an efficacy signal and to assess safety. A larger phase 3 trial is required and underway to generate results that might be more generalizable to the broader renal transplant population.

There were no clear adverse safety signals in the ANG-3777 arm. There were no deaths or discontinuations because of the study drug. A similar proportion of subjects in the ANG-3777 and placebo arms experienced AEs (89.5% versus 88.9%), TEAEs (78.9% versus 88.9%), and SAEs/TESAEs (42.1% versus 44.4%). The number of AEs, TEAEs, and SAEs/TESAEs per subject were twice as high in the placebo arm versus the ANG-3777 arm. On the other hand, all 6 TEAEs adjudicated as possibly related to study drug occurred in the ANG-3777 arm, and there were 11 severe TEAEs in 6 subjects in the ANG-3777 arm versus 1 in the placebo arm, although no TESAEs were adjudicated as related to study drug. Events within individual SOCs were generally higher in the placebo arm. These safety results should be viewed in context: subjects in this study were transitioning from dialysis to transplantation, and the AEs observed were expected in this population. Overall, there was no clear pattern of differences suggesting an adverse safety signal for ANG-3777.

In this phase 2 trial, there was a signal for improved renal function in subjects treated with ANG-3777 relative to placebo, which manifested across laboratory and clinical treatment measures and was durable to 12 months. ANG-3777 had similar safety to placebo. These data support conducting a larger, well-controlled trial to further evaluate the safety and efficacy of ANG-3777 in patients with signs of kidney injury immediately following kidney transplantation.

## ACKNOWLEDGMENTS

We would like to acknowledge the contribution of Barry J. Browne, MD, who passed away before publication of this manuscript. Dr. Browne was a site lead investigator who enrolled patients, supported their care, and helped bring this trial to fruition. His support of this work was, and is, greatly appreciated by his colleagues.

## Supplementary Material


