# Supplementary material for: Renal Function Improvement Following ANG-3777 Treatment in Patients at High Risk for Delayed Graft Function After Kidney Transplantation
Source: Transplantation. 2021 Jan 26;105(2):443–50. doi: 10.1097/TP.0000000000003255 (PMC7837751; doi:10.1097/TP.0000000000003255)
Supplement: Supplementary file 1 [file tp-105-443-s001.pdf]

SDC1, Materials and Methods. Complete Inclusion and Exclusion Criteria

Inclusion criteria:

1. Males and females ≥18 years of age
2. Renal transplantation due to end-stage renal disease requiring chronic dialysis
3. Study drug could be administered ≤36 hours after transplantation
4. Received kidney from a healthy donor, with/without history of diabetes mellitus/ hypertension
5. Donor terminal SCr ≤2.2 mg/dL
6. Urine output <50 cc/hour over ≥8 consecutive hours; or creatinine reduction ratio <30% from pretransplantation to 24 hours posttransplantation
7. Reason for low urine output unlikely due to structural changes as determined by imaging with Doppler ultrasound
8. Dry weight ≤120 kg, body mass index (BMI) <35
9. Women of childbearing potential: negative serum pregnancy test before transplantation;
   agreed to use 2 forms of an effective birth control regimen, including barrier method, during the 28-day study period. Men agreed to use condoms during this period.
10. Subject capable of understanding and complying with the protocol
11. Subjects signed the informed consent document prior to performance of any study-related procedure, including screening

Exclusion criteria:

1. Preemptive renal transplantation

2. Signs and symptoms of volume depletion
3. Multiple organ transplantation
4. Recipient of pediatric en-bloc kidney transplantation
5. Cold ischemia time >40 hours
6. Measurable donor-specific antibody or positive crossmatch requiring deviation from standard immunosuppressive therapy
7. Pre-enrollment participation in an investigational drug or medical device study within 30 days or 5 half-lives, whichever was longer
8. Concurrent sepsis or active bacterial infection
9. Active malignancy or history of solid, metastatic or hematologic malignancy, except basal or squamous cell carcinoma of the skin that had been removed
10. Women of childbearing potential who were breast feeding
11. Positive human immunodeficiency virus (HIV) test
12. History of rheumatoid arthritis
13. Subjects requiring cytochrome P450 1A2 (CYP1A2) inhibitors (ciprofloxacin and/or fluvoxamine)
14. Subject unwilling or unable to comply with the protocol or to cooperate fully
15. Subject deemed medically unstable for the study

Table S1. Treatment-emergent Adverse Events by System Organ Class.

System Organ Class<br>Preferred Term	ANG-3777<br>(N = 19)	Placebo<br>(N = 9)
**Treatment-emergent adverse events**	**15 (78.9)**	**8 (88.9)**
**General disorders and administration site conditions**	**7 (36.8)**	**6 (66.7)**
Edema	2 (10.5)	3 (33.3)
Chest pain	0 (0.0)	3 (33.3)
Edema peripheral	2 (10.5)	1 (11.1)
Asthenia	0 (0.0)	2 (22.2)
**Metabolism and nutrition disorders**	**7 (36.8)**	**3 (33.3)**
Hypomagnesaemia	3 (15.8)	2 (22.2)
Hypocalcemia	3 (15.8)	0 (0.0)
Hyperkalemia	1 (5.3)	1 (11.1)
Hypokalemia	1 (5.3)	1 (11.1)
Hyponatremia	2 (10.5)	0 (0.0)
Hypophosphatemia	2 (10.5)	0 (0.0)
**Gastrointestinal disorders**	**6 (31.6)**	**3 (33.3)**
Constipation	4 (21.1)	2 (22.2)
Diarrhea	0 (0.0)	2 (22.2)
Nausea	2 (10.5)	0 (0.0)
**Renal and urinary disorders**	**6 (31.6)**	**2 (22.2)**
Nocturia	2 (10.5)	0 (0.0)
Renal failure acute	0 (0.0)	2 (22.2)
**Vascular disorders**	**4 (21.1)**	**3 (33.3)**
Hypotension	1 (5.3)	2 (22.2)
**Respiratory, thoracic and mediastinal disorders**	**1 (5.3)**	**4 (44.4)**
Dyspnea	0 (0.0)	3 (33.3)
**Cardiac disorders**	2 (10.5)	2 (22.2)
Cardiac failure congestive	1 (5.3)	1 (11.1)
**Reproductive system and breast disorders**	**2 (10.5)**	**3 (33.3)**
Scrotal edema	1 (5.3)	2 (22.2)
Penile pain	1 (5.3)	1 (11.1)
**Blood and lymphatic system disorders**	**3 (15.8)**	**1 (11.1)**
Anemia	2 (10.5)	0 (0.0)
**Nervous system disorders**	**2 (10.5)**	**2 (22.2)**
Dizziness	1 (5.3)	1 (11.1)
Tremor	1 (5.3)	1 (11.1)
**Surgical and medical procedures**	**2 (10.5)**	**2 (22.2)**
Wound drainage	1 (5.3)	1 (11.1)
**Psychiatric disorders**	**1 (5.3)**	**2 (22.2)**
Insomnia	1 (5.3)	1 (11.1)

Table S2. Treatment-emergent Serious Adverse Events by System Organ Class.

System Organ Class<br>Preferred Term	ANG-3777<br>(N = 19)	Placebo<br>(N = 9)
**Treatment-emergent serious adverse events**	**8 (42.1)**	**4 (44.4)**
**Renal and urinary disorders**	**3 (15.8)**	**2 (22.2)**
Renal failure acute	0 (0.0)	2 (22.2)
Renal necrosis	1 (5.3)	0 (0.0)
Tubulointerstitial nephritis	1 (5.3)	0 (0.0)
Ureteral necrosis	1 (5.3)	0 (0.0)
Ureteric stenosis	1 (5.3)	0 (0.0)
Urinoma	1 (5.3)	0 (0.0)
**Infections and infestations**	**1 (5.3)**	**3 (33.3)**
Atypical pneumonia	0 (0.0)	1 (11.1)
Pneumonia	0 (0.0)	1 (11.1)
Urinary tract infection	0 (0.0)	1 (11.1)
Urosepsis	1 (5.3)	0 (0.0)
**Vascular disorders**	**3 (15.8)**	**1 (11.1)**
Arteriovenous fistula	1 (5.3)	0 (0.0)
Hematoma	1 (5.3)	0 (0.0)
Hypertension	0 (0.0)	1 (11.1)
Lymphocele	1 (5.3)	0 (0.0)
**Cardiac disorders**	**2 (10.5)**	**1 (11.1)**
Cardiac failure congestive	1 (5.3)	1 (11.1)
Cardiac failure	1 (5.3)	0 (0.0)
**General disorders and administration site conditions**	**1 (5.3)**	**2 (22.2)**
Chest pain	0 (0.0)	1 (11.1)
Implant site extravasation	0 (0.0)	1 (11.1)
Medical device complication	1 (5.3)	0 (0.0)
**Injury, poisoning and procedural complications**	**1 (5.3)**	**1 (11.1)**
Incision site pain	1 (5.3)	0 (0.0)
Transplant dysfunction	0 (0.0)	1 (11.1)
Wound	0 (0.0)	1 (11.1)
**Metabolism and nutrition disorders**	**2 (10.5)**	**0 (0.0)**
Dehydration	1 (5.3)	0 (0.0)
Hypocalcemia	1 (5.3)	0 (0.0)
**Respiratory, thoracic and mediastinal disorders**	**0 (0.0)**	**2 (22.2)**
Pulmonary hypertension	0 (0.0)	1 (11.1)
Respiratory failure	0 (0.0)	1 (11.1)
**Gastrointestinal disorders**	**1 (5.3)**	**0 (0.0)**
Umbilical hernia	1 (5.3)	0 (0.0)
**Investigations**	**0 (0.0)**	**1 (11.1)**
Blood creatinine increased	0 (0.0)	1 (11.1)
**Nervous system disorders**	**0 (0.0)**	**1 (11.1)**
Metabolic encephalopathy	0 (0.0)	1 (11.1)

**System Organ Class**<br>Preferred Term	**ANG-3777**<br>**(N = 19)**	**Placebo**<br>**(N = 9)**
**Surgical and medical procedures**	**0 (0.0)**	**1 (11.1)**
Incisional hernia repair	0 (0.0)	1 (11.1)
